# What is lost when training goes digital?

**DOI:** 10.1002/hem3.148

**Published:** 2024-08-08

**Authors:** Tanya Freeman, Stephen P. Hibbs

**Affiliations:** ^1^ Barts Health NHS Trust London UK; ^2^ Wolfson Institute of Population Health Queen Mary University of London UK

Haematology professionals in training have a world of learning at their fingertips. Well‐written textbooks and review articles are joined by YouTube channels, Twitter accounts, and data interpretation banks. Professional haematology organisations have invested in digital education, providing extensive repositories of podcasts, image banks, and recorded lecture series. Regional and national postgraduate haematology training days and haematology conferences can now be ‘attended’ through a laptop and an internet connection.

There are clear benefits of digital repositories and remote meeting software for both trainers and trainees. With so many resources to choose from, trainees can direct their own learning through whatever style works best for them^1^ and learn at their own pace. Accessibility is increased: a trainer gives a single lecture for a ‘live’ group, and the recording can be used by trainees who are unwell, who work part‐time, or who have clinical commitments during the learning session. Virtual and prerecorded teaching can be used at scale and reduces costs. For international or distant meetings, virtual attendance has a much smaller carbon footprint.

The COVID‐19 pandemic accelerated the transition to digital and asynchronous training which may be coagulating to a ‘new normal’. In this article, we argue that this transition should be questioned by presenting five overlooked benefits of in‐person, synchronous (‘live’) haematology training.

First, a face‐to‐face fixed training commitment provides a protected space and time and a positive social pressure to engage *now*. Training is often ‘protected’ from clinical commitments, and social etiquette requires enough trainees to be present to avoid embarrassing the trainer. In contrast, virtual sessions tempt trainees to attempt to multitask or defer the learning opportunity altogether, imagining that they can watch the recording later. However, the session recording may join the ever‐increasing pile of things we might one day listen to or read—but probably won't get round to.

Second, live sessions provide a clear and immediate focus for learning. As digital educational repositories proliferate, the amount of available educational material can become overwhelming. How do you decide what to look at or listen to? Is a particular learning resource still up to date? In contrast, live sessions provide focus and a clear agenda for learning. Trainers can highlight major updates and curate the most valuable reading and educational resources to refer to before or after the live session. Interactive sessions can also reveal to trainees the topics that are ‘unknown unknowns’: gaps in their knowledge that they didn't know they needed to know.

Third, face‐to‐face training helps trainees to learn from each other. Interacting and connecting with others is essential for learning individually and collectively.^2^ Different ‘learning environments’ help or hinder connections between learners, teachers, and the material being studied. Successful learning is influenced more by the quality of learning environment than the ability and motivation of individual learners.^3^ Picture the traditional learning environment for morphology: trainees sitting together around a multiheader microscope guided by an experienced morphologist. In this deceptively simple environment, trainees inspect a blood film, form observations and interpretations, practice sharing their tentative conclusions verbally, compare these with their peers and trainers, refine their interpretations, and develop a sense of subjectivity. Now picture an individual trainee viewing the same blood film through an online morphology bank: which of these skills are practiced? Even if there is the option to practice interpretation, it is challenging to do so meaningfully when the answer is one click away. Furthermore, ‘The Answer’ on an image bank can be misleadingly definitive, oversimplifying the real world of nuance and uncertainty. These two learning environments are not interchangeable.

Fourth, face‐to‐face training helps trainees develop their professional identity. Postgraduate medical training builds clinical and practical skills but also develops an individual's own identity as a professional.^4^ Local groups of haematology trainees form ‘communities of practice’ as they learn and train together.^5^, ^6^ They can compare themselves to their peers which can be motivating; ‘I want to be more like them’ (and conversely, at times this comparison can be demotivating). When trainees join a community of practice, they become more skilled in the tasks and language of their profession—crucial for developing professional identity. Speaking about uncertainty, sadness, or other vulnerable experiences is important for a healthy professional identity but requires the trust and intimacy that face‐to‐face teaching fosters. In addition, the times before and after the main teaching event provide an opportunity to catch up and deal with small items of clinical conversation. These interactions are a key part of developing professional identity and trust; they occur unobtrusively when meeting face‐to‐face.

Finally, we should consider trainee wellbeing. Face‐to‐face training is generally more enjoyable than meeting online. When we meet in person, we can enjoy the company of each other, someone making a joke, someone bringing in food, and someone making the coffee. This enjoyment of time together while learning is intrinsically good and helps sustain us through the more difficult parts of the job.

Perhaps, the real question is to ask what we are trying to achieve during haematology training. Recorded lectures, image banks, and virtual meetings may well be sufficient to attain some learning goals, particularly where these are focused on building knowledge. Digital repositories are excellent resources to prepare for postgraduate exams, and self‐directed learning is an important skill to develop throughout the span of a career. But we also need spaces where professional identity is built and shaped, trust between colleagues is developed, and where we take time to enjoy the company of one another. Face‐to‐face training has been critical to these goals throughout the history of medical training and remains worth preserving in an increasingly digital and asynchronous world.

## AUTHOR CONTRIBUTIONS

Both authors conceptualised the article. Tanya Freeman wrote the initial draft. Stephen P. Hibbs critically reviewed the article and revised it. Both authors agreed to the final version.

## CONFLICT OF INTEREST STATEMENT

The authors declare no conflict of interest.

## FUNDING

Tanya Freeman is supported by a teaching fellowship funded by the Royal College of Pathologists and Barts Health NHS Trust. Stephen P. Hibbs is supported by a HARP doctoral research fellowship, funded by the Wellcome Trust (Grant number 223500/Z/21/Z). No funding was received for this publication.

## Data Availability

Data sharing not applicable to this article as no data sets were generated or analysed during the current study.
